# Comparative verification of control methodology for robotic interventional neuroradiology procedures

**DOI:** 10.1007/s11548-023-02991-2

**Published:** 2023-07-17

**Authors:** Benjamin Jackson, William Crinnion, Mikel De Iturrate Reyzabal, Harry Robertshaw, Christos Bergeles, Kawal Rhode, Thomas Booth

**Affiliations:** 1 Biomedical Engineering and Imaging Sciences, Kings College London, 1 Lambeth Palace Rd, London, SE1 7EU UK; 2 Department of Neuroradiology, Kings College Hospital, Ruskin Wing, London, SE5 9RS UK

**Keywords:** Thrombectomy, Robotics, Interventional neuroradiology, Haptics

## Abstract

**Purpose:**

The use of robotics is emerging for performing interventional radiology procedures. Robots in interventional radiology are typically controlled using button presses and joystick movements. This study identified how different human–robot interfaces affect endovascular surgical performance using interventional radiology simulations.

**Methods:**

Nine participants performed a navigation task on an interventional radiology simulator with three different human–computer interfaces. Using Simulation Open Framework Architecture we developed a simulation profile of vessels, catheters and guidewires. We designed and manufactured a bespoke haptic interventional radiology controller for robotic systems to control the simulation. Metrics including time taken for navigation, number of incorrect catheterisations, number of catheter and guidewire prolapses and forces applied to vessel walls were measured and used to characterise the interfaces. Finally, participants responded to a questionnaire to evaluate the perception of the controllers.

**Results:**

Time taken for navigation, number of incorrect catheterisations and the number of catheter and guidewire prolapses, showed that the device-mimicking controller is better suited for controlling interventional neuroradiology procedures over joystick control approaches. Qualitative metrics also showed that interventional radiologists prefer a device-mimicking controller approach over a joystick approach.

**Conclusion:**

Of the four metrics used to compare and contrast the human–robot interfaces, three conclusively showed that a device-mimicking controller was better suited for controlling interventional neuroradiology robotics.

## Introduction

Stroke is the second leading cause of death across the world, annually killing approximately 6 million people. The time from onset to treatment is known to be particularly critical, with the effectiveness of the treatment declining rapidly and significantly in the first few hours after stroke [1]. More recently, mechanical thrombectomy (MT) has shown substantially improved clinical outcomes in patients with large vessel occlusion with acute ischaemic stroke [2, 3]. MT may be effective up to 6 h after stroke onset, with evidence for intervention up to 24 h after stroke onset in selected patients. However, evidence also demonstrates a reduction in the effectiveness of MT with increasing time from stroke onset [4]. The proportion of patients eligible for MT in the UK has been consistently estimated at 10% [5], however, only about 10% of eligible patients receive MT [6] as providing MT presents a significant logistical challenge for health services.

Several groups have recently described robotically-assisted cerebral angiography globally [7–10]. For neurointerventional applications, the lack of a platform specifically designed to accommodate small micro-catheters and micro-guidewires and the technically demanding micro-movements required to successfully navigate these tools through the cerebral vasculature, has left neurointerventional robotics less frequently explored then other interventional specialties [9].

Realising robotic systems that can accurately control neurointerventional catheters and guidewires can drastically improve the ratio of patients receiving MT. A tele-operated system would allow patients to receive stroke care from any available neurointervention centre [11]. As long as the patient was within close proximity to the responder robotic system, an active neurointerventionist from any geographic location could connect and perform the procedure remotely.

Two basic mechanisms have been described for controlling robotic surgery in general. Some robotic systems have device-mimicking controllers, essentially directly copying the operator movements. Other systems transform the movements of the operator such that a joystick, for example, can manipulate the catheter and guidewire [7–10]. Additional systems can be considered for robotic systems, such as delivering complementary feedback to the interventionist or using autonomous systems to assist the neurointerventionist. Little evidence has been provided to demonstrate which robotic control method provides the best clinical outcomes, if any. Our study aims to identify how different human–robot interfaces affect endovascular surgical performance using interventional neuroradiology simulations.

## Materials and methods

### Software-based medical simulation

For this project, Simulation Open Framework Architecture (SOFA) was chosen to handle the soft body simulation [12]. SOFA is an open source platform for the mechanical simulation of multiple parameters. The framework is primarily targeted at real-time simulation with an emphasis on medical simulation.

Our simulation had four requirements to address during its design: (1) vessel anatomy, (2) vessel mechanical simulation, (3) catheter and guidewire beam modelling and (4) optimisation.

**Fig. 1:**
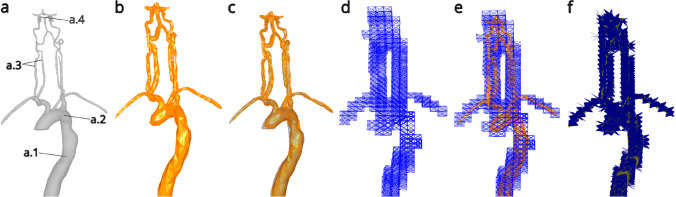
**a** The visual model used for rendering the vessels. (a.1) The descending aorta. (a.2) The aortic arch. (a.3) The carotid and vertebral arteries. (a.4) The circle of Willis and branching vessels. **b** The collision mesh of the vessels used for calculating the forces at a point of contact. **c** The collision model and the visual model. **d** The spring force field used for computing the mechanical deformation of the vessels (stiffness = 30,000). **e** The spring force field and the collision model. **f** A visualisation of the barycentric mapping

- *Vessel anatomy* Vessel models were generated using two computed tomography angiography (CTA) scans. One scan ranged from the aortic arch to the cerebral vessels (including carotids and vertebral arteries). The other scan was of the abdominal and thoracic regions capturing the femoral arteries, descending aorta and also the aortic arch. These scans were separately processed into surface meshes (850 vertices) and manually combined to create one continuous model.
- *Vessel mechanical simulation* High fidelity mechanical simulation caused significant computation challenges (using an Intel$\circledR$ Core$^{\textrm{TM}}$ i7-7700 Processor, 16 GB RAM, NVIDIA Quadro M4000 GPU). The most realistic method for generating mechanical soft body vessel simulation is to directly animate the vessel surface mesh with SOFA forcefield components [12]. However, as the number of triangles in the surface mesh increases, the computational cost increases exponentially. Instead, a grid was generated in the shape of the vessel mesh, the grid was barycentrically mapped back to the vessel mesh and the grid was simulated with a spring force field SOFA component (Fig. 1).
- *Catheter and guidewire beam modelling* Simulation of the catheters (160 vertices at 47,000,000 Young’s modulus) and guidewires (120 vertices at 43,000,000 Young’s modulus) was implemented using the Beam Adapter plugin for SOFA. The Beam Adapter plugin used the product information of the catheters and guidewires in Table 1 to generate the resting topology and the mechanical properties in the simulation.
- *Optimisation* The number of collisions in a scene will significantly effect computational cost. As our results relied on the contact forces throughout the simulation, reducing the number of contact points could not be done extensively without risking the integrity of the data. However, there are still methods to reduce the number of collisions for neurointerventional procedures. Typically, neurointerventional procedures occur in two main steps. First, navigation was performed from the femoral artery entry to the large neck vessels with a large catheter (for example, a 0.088 inch Neuron Max, Penumbra). Once this guide catheter was in place, it remained there while smaller neurointervention catheters and wires were passed through it to complete the rest of the navigation. This stepwise approach was similarly used to optimise the simulation. Once the large catheter was in place, it was locked in software, reducing the number of collision calculations to zero. The rest of the navigation could then be completed in real time, with new collisions recorded during this second step.

**Table 1:** The catheters and guidewires used for the navigation

Name	Type	Shape	Supplier
Penumbra SIM	Catheter	SIM$^\textrm{b}$	Penumbra, California, USA
Terumo 0.035	Guidewire	MPA$^\textrm{a}$	Terumo, Tokyo, Japan
Echelon 10 (1.7 F)	Micro-catheter	Straight	Medtronic, Minnesota, USA
Synchro 14	Micro-guidewire	MPA$^\textrm{a}$	Stryker NeuroVascular, Cork, Ireland

$$^\textrm{a}$$Multipurpose angiographic

$$^\textrm{b}$$Simmons

### Controllers

Generally, two operating principles are used to control robotic endovascular systems, a device-mimicking control approach and a joystick approach. Haptic and non-haptic controllers have been described by Yin et al. [13] with a maximum force deviation of 0.012 N at 6.1 mm/s using magnetorheological fluids which, while accurate, is expensive, Shi et al. [14] with an average force deviation of 0.027 N at 20 mm/s using electromagnetic force generator which again, while accurate, is expensive and Jia et al. [15] with a maximum force deviation of 1.42 N at 3.66 mm/s using a Phantom Omni, Delft, Netherlands which uses off the shelf components that are more easily accessible but with reduced force accuracy. This experiment included three controllers that broadly cover the spectrum of control methodology.

**Fig. 2:**
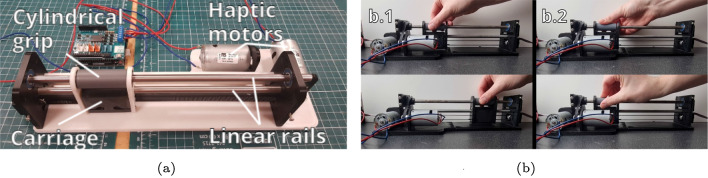
**a** Robotic haptic controller used for controlling interventional radiology robotics. **b** (b.1) Advancing and retracting the catheter or guidewire with the controller (2) Rotating the catheter or guidewire with the controller

- *Joystick controller* The joystick on the controller was used to advance, retract and rotate the controlled catheter and guidewire. Buttons allowed the user to choose between controlling the catheter and guidewire.
- *Robotic controller—haptics off* (Fig. 2)—The robotic controller (RC) had 2 degree of freedom (DoF). The user held the cylindrical grip (Fig. 2). The first DoF slid the carriage across the linear rails (Fig. 2a, b) controlling the advancing and retracting DoF of the catheters and guidewires. The cylindrical grip could be rotated to control the rotation DoF of the catheter and guidewire. Rotary encoders are attached to the linear and rotary axis to measure the position of the controller. To operate the controller beyond its stroke length, a push to make switch was used to disconnect the controller from the simulation. This allowed the user to manually reset the controller ready for the next manoeuvre. Two more switches were used to select the controlled catheter or guidewire.
- *Robotic controller—haptics on* (Fig. 2). The RC was used with the haptic feedback turned on. Forces from the distal tip of the catheter and guidewire were calculated in the simulation scene Materials and methods section. Haptic feedback was individually applied to each DoF using the haptic motors (Mellor FRS-380SEM) (Fig. 2).

### Experiment

The study was conducted with six experienced (greater than five years clinical experience) interventional neuroradiologists (UK consultant grade; US attending equivalent) and two novice (less than three years clinical experience) interventional neuroradiologists and one interventional radiologist (UK specialist trainee grade; US fellow equivalent). Participants were given four minutes of practise to familiarise themselves with the system, two minutes of practise with the joystick style controller in Sect. 2.2 and two minutes of practise with the haptics off (RC (off)) in Sect. 2.2.

**Fig. 3:**
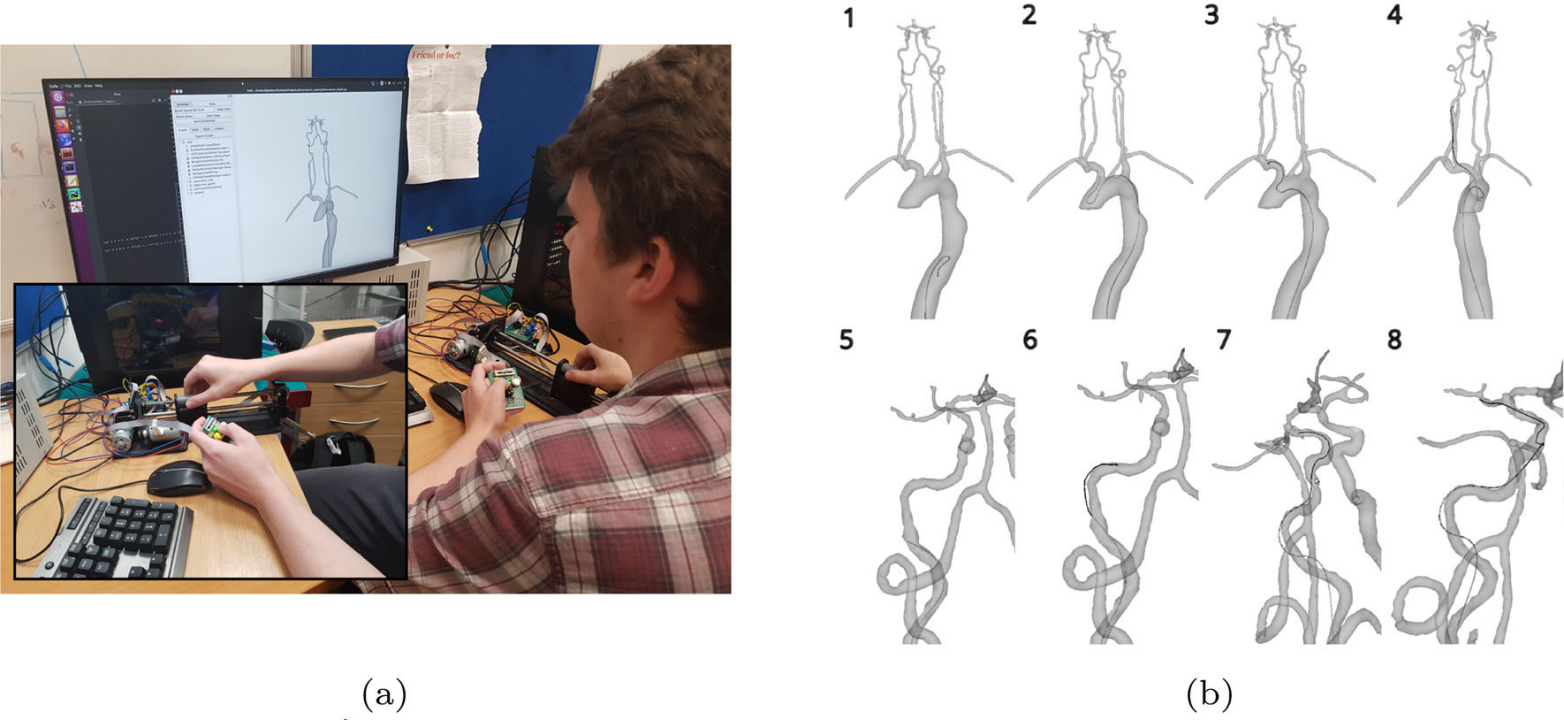
**a** A participant completing the experiment using the RC, buttons are used to select control of the catheter or guidewire and to disconnect the controller from the simulation. **b** A chronological example of the navigation completed by the participant

Following this, the participants were asked to complete three identical navigations with the three controllers in a randomised order. One navigation was performed using the RC (off) (Fig. 3), one was performed using the RC (off) and one performed using the joystick controller.

The navigation began in the descending aorta. The participants used a simmons (SIM)-shaped guide catheter and a multipurpose angiographic (MPA) guidewire to navigate into the brachiocephalic artery. The guidewire was then navigated into the common carotid artery and the internal carotid artery (ICA) (Fig. 3b, 1–4). At this stage, the simulation was paused and the participants changed to the micro-guidewire and micro-catheter used for navigating cerebral arteries. The micro-catheter and micro-guidewire were used to navigate to the middle cerebral artery (MCA) where the navigation was complete (Fig. 3b, 5–8).

#### Formative evaluation

Quantitative data, including the time taken for the procedure (primary outcome), the force exerted on the vessel wall, the number of incorrect vessel catheterisations and the number of prolapses, was extracted from the simulation profile. Qualitative data was recorded in the form of a questionnaire after the experiment had concluded.

*Time taken—primary outcome*

The primary outcome of the study was the time taken to complete the procedure. In the operating room, a faster procedure has several benefits: reduced fatigue on the operator, a reduced time the patient is under anaesthetic, a higher patient throughput and a decreased risk of thromboembolic complications. In the context of MT, this is particularly important to minimise the ischaemic damage to the brain. Separate procedural sections based on anatomy were recorded additionally for subgroup analysis that consisted of:

- Descending aorta to brachiocephalic artery.
- Brachiocephalic artery to internal carotid artery (20 mm past the carotid bifurcation).
- Carotid bifurcation to distal M1.

*Vessel wall forces—secondary outcome 2a*

The forces from the simulation at any contact point between the vessel wall and the catheter and guidewire were used for analysis. Mean forces and forces exceeding certain levels were analysed.

During endovascular procedures, a concern is puncturing a vessel wall causing a perforation. Using Saito et al. [16] value of 150 mN to puncture the vein wall of a white rabbit and applying a safety factor of 10, we can assign a safety threshold of 15 mN as maximum desirable target. However, this is likelt to be an overly cautious estimate as (1) it is plausible that the force required to puncture a vein is far less than to puncture an artery which is under higher blood pressure physiologically and (2) that the force required to puncture an artery in humans is higher than the force required to puncture an artery in rabbits. Therefore, whilst we assign a safety threshold of 15 mN as maximum desirable target, we estimate that the plausible puncture force of human arteries is 10 times the puncture force in rabbit veins, and we assign a safety threshold of 1500 mN as an absolute essential target requirement for successful navigation.

*Incorrect vessel catheterisations—secondary outcome 2b*

**Fig. 4:**
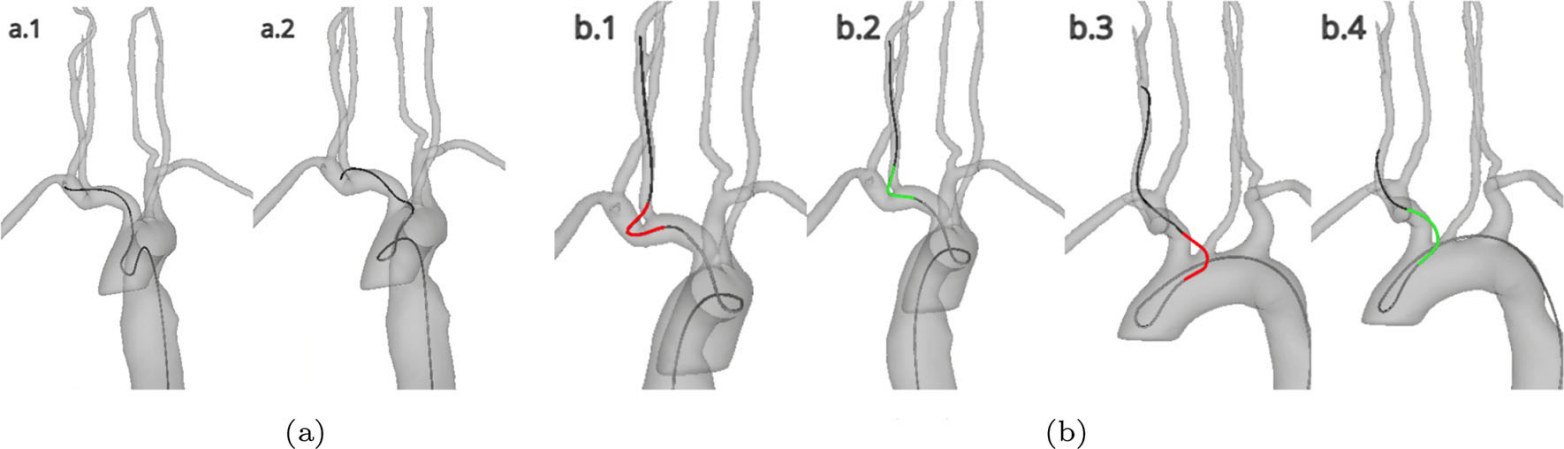
**a** (a.1) The guidewire inserting into the subclavian artery by 10 mm. (a.2) The guidewire retracting to under 5 mm insertion into the vertebral artery. **b** (b.1) The guidewire prolapsing out of the common carotid artery by 10 mm. (b.2) The guidewire retracting to under a 5 mm prolapse in the common carotid artery. (b.3) The guidewire prolapsing out of the brachiocephalic artery by 10 mm. (b.4) The guidewire retracting to under a 5 mm prolapse of the brachiocephalic artery

Incorrect catheterisation was defined as the proximal tip of the catheter and guidewire extending > 10 mm into a vessel that had been inadvertently selected during the planned route. In all cases the route selected was: descending aorta–brachiocephalic–common carotid–internal carotid–middle cerebral artery (distal M1). If at any point the catheter or guidewire was navigated over 10 mm into a vessel not on this route, the count of incorrect vessel catheterisations was incremented. The count could not be incremented again until the catheter or guidewire had been retracted to under 5 mm within the incorrect vessel lumen (Fig. 4).

*Catheter and guidewire prolapses—secondary outcome 2c* A prolapse occurs most frequently when the tip of the guidewire is caught in the vessel lumen causing it to buckle. In the simulation, a prolapse was defined as the catheter or guidewire falling out of a vessel by more than 10 mm (Fig. 4b). For each simulation, if a catheter or guidewire prolapse was encountered, the count was incremented and could not be incremented again until the prolapse was recovered. The recovery of a prolapse was defined as the prolapsed catheter or guidewire being retracted to under 5 mm (Fig. 4b).

## Results

The evaluation of the system was performed in the Neuroradiology Department at Kings College Hospital (KCH), London, UK. Nine interventional neuroradiologists with different levels of experience performed a simulated interventional neuroradiology navigation. Three of the participants presented themselves as novice and six presented themselves as experts (more than 5 years of interventional neuroradiology experience)

### Primary outcome: time taken to navigate

**Fig. 5:**
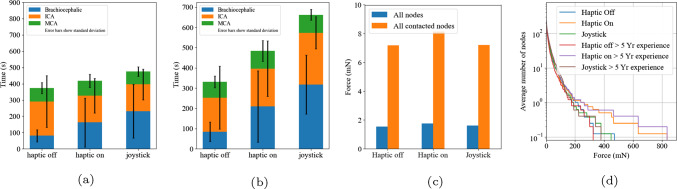
**a** The mean time taken for all participants to navigate. **b** The mean time taken for participants with greater than 5 years interventional neuroradiology experience to navigate. **c** The mean of the maximum force each node of the vessel collision mesh experienced during the navigation. **d** The number of nodes that experienced a force over *N* during the navigation

Figure 5 shows the mean completion time over the nine participants. The RC (off), the RC (on), and the joystick controller had a completion time mean of 373.58 s, 397.84 s and 414.04 s respectively (*P* = 0.765, analysis of variance (ANOVA)). When analysing participants with greater the five years experience, the completion time mean was 330.71 s, 446.30 s and 508.76 s respectively (*P* = 0.027, ANOVA)).

### Secondary outcome 2a: force at the vessel wall

Figure 5c shows the mean maximum force at every node on the collision mesh and the mean maximum force at every node on the mesh that experienced a collision. The mean maximum force at every node for the RC (off), the RC (on) and the joystick controller was 1.557 mN, 1.760 mN and 1.611 mN (*P* = 0.768, ANOVA), respectively. When removing nodes that did not experience a collision during the navigation the mean was 7.189 mN, 8.157 mN and 7.216 mN (*P* = 0.724, ANOVA), respectively.

The number of nodes with a force greater than 15 mN that the RC (off), the RC (on) and the joystick controller exerted on the vessel wall was 62.00, 68.75 and 63.00, respectively (Fig. 5d). With a threshold of 150 mN, the average number of nodes was 2.38, 2.25 and 1.75, respectively. With a threshold of 300 mN, the mean number of nodes was 0.25, 0.75 and 0.38, respectively. In the expert group, there are two collisions that exceed 600 mN, both from the same participant. All of the collisions are below the plausible vessel rupture value of 1500 mN.

### Secondary outcome 2b: number of incorrect vessel catheterisations

**Fig. 6:**
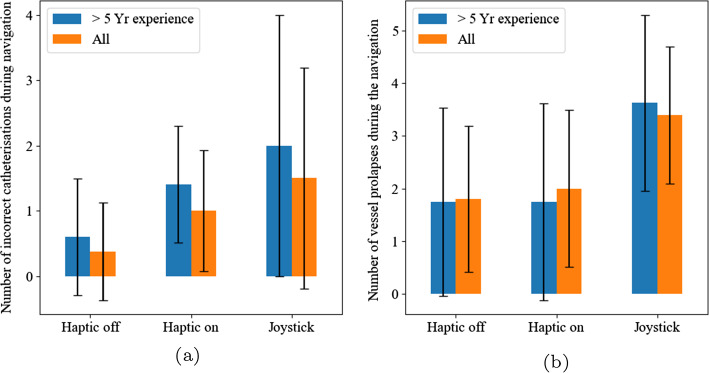
**a** The mean number of incorrect vessel catheterisations during the navigation. **b** The mean number of prolapses during the navigation

Incorrect catheterisation was defined as the proximal tip of the catheter and guidewire extending > 10 mm into a vessel that had been inadvertently selected during the planned route. In all cases the route selected was: descending aorta–brachiocephalic–common carotid–internal carotid–middle cerebral artery (distal M1). If at any point the catheter or guidewire was navigated over 10 mm into a vessel not on this route, the count of incorrect vessel catheterisations was incremented. The count could not be incremented again until the catheter or guidewire had been retracted to under 5 mm within the incorrect vessel lumen (Fig. 4).

Figure 6 shows the mean number of incorrect catheterisations for each participant during the navigation. The mean across all participants for the RC (off), the RC (on) and the joystick controller was 0.38, 1.00 and 1.50 [*P* = 0.192, RC (off), the RC (on)] respectively. When only analysing participants with greater than 5 years interventional neuroradiology experience the mean increases slightly to 0.6, 1.4 and 2.0 [*P* = 0.303, RC (off), the RC (on)] respectively. This trend of a difference [*P* = 0.052, RC (off), the RC (on)] between the novice and experienced groups was unexpected.

### Secondary outcome 2c: number of catheter and guidewire prolapses

Figure 6b shows the number of prolapses for the RC (off), the RC (off), remained the same at 1.8 and 2.0 mean prolapses per navigation, respectively. However, when using the joystick controller, participants prolapsed the vessel significantly more often at 3.4 (*P* = 0.019, ANOVA) prolapses per navigation. When comparing radiologists with more than 5 years experience, the mean number of prolapses between the RC (off), the RC (off) and the joystick controller was 1.75, 1.75 and 3.63 (*P* = 0.335, ANOVA) respectively. The novice group and the expert group experienced the same number of prolapses (*P* = 0.924, ANOVA).

### Qualitative results

**Fig. 7:**
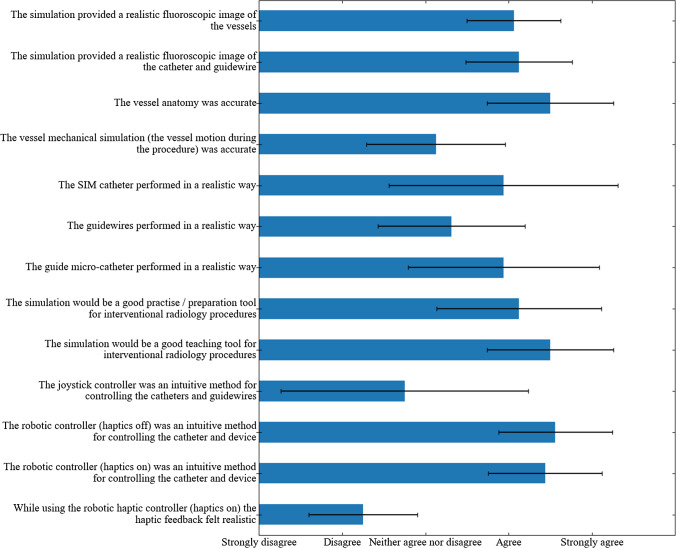
The results from the participant survey post-navigation. The mean score and the standard deviation is shown for each question

Figure 7 shows the qualitative results that were obtained in the form of a questionnaire after the participants completed the navigations. The survey employed the 5 point Likert scale. The questions were targeted to different aspects of the experiments, including: *Realism* (greater than 5 years experience only)—the degree to which the simulation authentically mimics real endovascular navigations. *Usefulness*—the degree to which the participants found the simulation a useful teaching or training tool. *Controllers*—which controller the operator found the most intuitive.

The participants agreed that the fluoroscopic image of the vessels and the fluoroscopic image of the catheter and guidewire were realistic (4.06—Agree and 4.13—Agree, respectively).

The perceived usefulness of the system received the best grading. The scores evaluating the system in terms of its practise/preparation and educational value were 4.13 (Agree) and 4.50 (Agree—Strongly agree) respectively. The participants valued the ability to use a tool such as this to preoperatively plan procedures and to teach students techniques or demonstrate particularly tortuous anatomies.

The joystick controller was generally evaluated as the least intuitive controller with a mean score of 2.75 (Neither agree nor disagree). The RC (off) and the RC (on) had significantly higher mean score of 4.56 and 4.43, respectively (*P* = 0.004, ANOVA). However, when the results were split into levels of expertise, the joystick controller was graded higher among the novice group at 4.00 (Agree). This can plausibly be attributed to the device-mimicking nature of the RC closely mimicking the movements exhibited during a procedure, where expert participants are able to rely heavily on their existing skill when using this controller.

The realism of the haptic feedback appears to be the weakest graded feature in the simulation (2.25—Disagree).

## Discussion and conclusion

### Summary of findings

The study found that device-mimicking control for robotic endovascular procedures had better outcomes than joystick style control. Three of the metrics showed that the RC is superior to the joystick controller while one metric showed that the control methodology was comparable. Qualitative data also showed that all participants preferred device-mimicking control methodology. The study also showed that haptic feedback may not be as valuable for endovascular robotics as initially thought. However, according to surveys, the haptic feedback did not appear very real, and further study is needed to confirm this assertion definitively.

### Comparison with studies worldwide

A study conducted by Gani et al. [17] used a virtual reality bone drilling simulation to show that haptic control is significantly better than non-haptic control for training. They suggest the implementation of haptics in surgical training simulations will improve their educational value. However, bone drilling procedures require higher forces than interventional neuroradiology procedures. Our results indicate that haptic feedback did not appear to be an important factor in interventional neuroradiology procedures.

### Study explanations and findings

The study examined the difference between device-mimicking control and joystick-based control for robotic endovascular procedures. The study used a simulated endovascular environment based in SOFA and three different control methods (Sect. 2.2). The force at the vessel wall metric showed no difference in controller type between mean force for all nodes and mean force of all nodes that experienced a collision. However, the other three metrics showed that the device-mimicking controller had advantages over the joystick controller. This is particularly true when analysing only participants with greater than five years interventional neuroradiology experience. This can be attributed to the device-mimicking nature of the RC that closely mimics the movements exhibited during the procedure. In this scenario, expert participants plausibly were able to rely heavily on their existing skill when using this controller. In addition, operators would likely practise for hundreds of hours and it is possible that they would become more proficient with joystick operation over time. It is also plausible that joystick operation reduces hand fatigue which, over an extended period of time, may become a significant factor in operator preference. It should also be noted that robotic catheters that can manipulate the tip such as the Swift Ninja, Utah, United States [18] may become more mainstream and the control methods in this paper may be affected. As expected, the expert group generally experienced the same number of catheter and guidewire prolapses during a procedure across all controllers (*P* = 0.924, ANOVA). However, although the results are non-significant, the trend suggests that this group incorrectly catheterised more vessels than the novice group (*P* = 0.052, ANOVA). Anecdotally, participants commented that the vessel anatomy was especially tortuous. In particular, the bifurcation of the common carotid artery from the brachiocephalic artery was difficult to see in the anteroposterior (AP) view. Experienced participants would attempt to catheterise the common carotid from the AP view more than novice participants. Most participants eventually switched to an obliqued view with novice participants switching view earlier in the procedure. For this reason, experienced participants generally had a higher number of incorrect catheterisations.

The force on the vessel wall did exceed Saito et al. [16] force measurements of 150 mN to puncture the vessel of a white rabbit with a 0.4 mm needle. Forces infrequently exceed 300 mN, double the value needed to puncture the vein wall in rabbits but plausibly a smaller force than would be required to puncture arteries in humans, as shown in Sect. 3.2. In the expert group, there were two nodes that exceeded 600 mN of force, both from the same participant using the RC (on).

All metrics indicated that the presence of haptic feedback had no statistically significant change in performance. This is an important discovery, as haptic feedback is often considered a useful tool, along with visual representation, to navigate the catheter and guidewire. As catheters and guidewires are often small and light, especially micro-catheters and micro-guidewires for cerebrovascular navigations, the haptic feedback is anecdotally not as important as the visual representation. However, haptic feedback may still be important in some scenarios. For example, during MT, when the thrombus is being retrieved, haptic feedback may be important. Alternatively, when a catheter or guidewire is caught or stuck in a vessel, haptic feedback may be important to prevent damage to the vessel lumen. Robotic endovascular systems may be able to employ cheaper, less sensitive force sensors that sense when a larger force is being imposed on the catheter and guidewire to accommodate these scenarios, while not necessarily being able to sense the microforces experienced during general navigation.

### Strengths and limitations

Computational limitations restrict the capacity of this study. The method used to animate the vessels needed to be simplified to achieve near real-time computation, these constraints led to the realism of the mechanical vessels being suboptimal.

A study containing more interventional neuroradiologists would be preferable. However, due to the limited amount of interventional neuroradiologists in the UK (less than 80) and the scope of this study, this was not feasible. Nonetheless, six senior interventional neuroradiologists represent 8% of the UK workforce which is a relatively high percentage.

### Unanswered questions and future directions

Further investigation is required to fully understand the impact of haptic feedback in endovascular procedures. This study appears to indicate that haptic feedback is not as important as once thought. However, we cannot make definitive conclusions as the qualitative assessment suggested that the haptic simulation may benefit from further refinement to appear more realistic. Particular scenarios such as MT may require haptic feedback to mitigate against extreme forces being applied by the operator.
